# Study on the Potential Mechanism of *Semen Strychni* against Myasthenia Gravis Based on Network Pharmacology and Molecular Docking with Experimental Verification

**DOI:** 10.1155/2022/3056802

**Published:** 2022-10-01

**Authors:** Pingfei Fang, Changwei Yu, Jian Liu, Gongying Deng, Min Zhang

**Affiliations:** ^1^Department of Pharmacy, The Second Xiangya Hospital, Central South University, Changsha 410011, China; ^2^Institute of Clinical Pharmacy, Central South University, Changsha 410011, China

## Abstract

**Background:**

*Semen Strychni* (SS) is an effective Chinese medicine formula for treating myasthenia gravis (MG) in clinics. Nonetheless, its molecular mechanism is largely unknown.

**Objective:**

Using network pharmacology, molecular docking, and experimental validation, we aim to identify the therapeutic effect of SS on MG and its underlying mechanism.

**Methods:**

The main ingredients of SS and their targets and potential disease targets for MG were extracted from public databases. The protein-protein interaction (PPI) network was constructed using the STRING 11.0 database, and Cytoscape was used to identify the hub targets. In addition, Gene Ontology (GO) and the Kyoto Encyclopedia of Genes and Genomes (KEGG) were used to identify molecular biological processes and signaling pathways. Then, AutoDock Via conducted molecular docking. The experimental autoimmune myasthenia gravis (EAMG) model in female Lewis rats, quantitative real-time polymerase chain reaction (qRT-PCR), Western blot, and enzyme-linked immunosorbent assay (ELISA) were performed to confirm the effect and mechanism of SS on MG.

**Results:**

The following active compounds and hub targets were identified by screening and analyzing: isobrucine, vomicine, (S)-stylopine, strychnine, brucine-N-oxide, brucine and AKT1, MAPK1, MAPK14, CHRM1, ACHE, and CHRNA4. KEGG enrichment analyses indicated that the cholinergic synapse and neuroactive ligand-receptor interaction signaling pathway may be necessary. The results of molecular docking revealed that the main active ingredients bind well to the hub targets. *In vivo* experiments proved that SS could improve the weight loss and Lennon scores in the EAMG model. Experiments in molecular biology showed that SS could treat MG by affecting the cholinergic synapse through the respective antibody, receptor, and key enzymes in the cholinergic pathway.

**Conclusion:**

This study provided a preliminary overview of the active constituents, primary targets, and potential pathways of SS against MG. SS ameliorated EAMG by regulating the cholinergic synaptic junction.

## 1. Introduction

Myasthenia gravis (MG) is a progressive autoimmune disorder caused by the production of antibodies against the acetylcholine receptor (AChR) at neuromuscular junctions (NMJ), resulting in fatigue and muscle weakness [1]. MG is considered to be a female-predominant disease, and its estimated annual prevalence rate is 2.19–36.71 per 100,000 people [2]. Although the pathogenesis of MG is poorly understood, the cholinergic pathway and inflammatory factors play a vital role in the development and progression of this disease. Authorities' most recent treatment recommendations include thymectomy, acetylcholine esterase inhibitors, corticosteroids, immunosuppressants, plasmapheresis, and intravenous immunoglobulins [3]. However, the most common treatments, pyridostigmine and corticosteroids, are associated with severe side effects due to their immunosuppressive properties, which patients may find intolerable. Consequently, numerous studies have investigated alternative MG treatments. Traditional Chinese medicine (TCM) have particular advantages in alleviating clinical symptoms, reducing recurrence, and enhancing the quality of life during the clinical treatment of MG [4, 5].

*Semen Strychni* (SS), the dried seeds of *Strychnos nux-vomica L. (Loganiaceae)*, is bitter and warm and, according to the TCM theory, belongs to the liver and spleen meridians. *SS* could be used to treat rheumatoid arthritis, facial nerve paralysis, and MG in folk [6]. Moreover, SS regulates inflammatory cytokines and reduces serum IL-1, IL-6, and TNF-*α* levels in rats with arthritis [7, 8]. In addition, SS ameliorates experimental autoimmune myasthenia gravis (EAMG) by decreasing acetylcholine receptor antibody (AChR-ab) levels and inhibiting the TLR-4/NF-kB signaling pathway in EAMG rats [9]. SS could also affect the neurotransmitter levels in the brain. Our colleagues discovered that SS could impact the monoamine oxidase content and metabolic pathways of tryptophan and dopamine, thereby altering neurotransmitters such as acetylcholine (ACH) levels in the brain [10]. Although the cholinergic pathway and inflammatory factors seem to participate in the mechanisms of SS against MG, the precise molecular mechanisms remain largely unknown.

Network pharmacology was first proposed by Hopkins in 2007, based on integrating multiple disciplines, including systems biology, network biology, molecular pharmacology, omics, and computational biology. Network pharmacology was widely utilized in the research of TCM as it focused on diseases from the holistic perspective of network balance and systematically explored the efficacy and mechanisms of TCM with multicomponent, multitarget, and multimechanism effects [11]. Molecular docking technology designs drugs based on the characteristics of receptors and their interactions with drug molecules [12]. It could be applied to investigate the binding ability of the active components to key targets.

This study investigated the potential targets and pathways of SS on MG using network pharmacology analysis. This method generated protein-protein interaction (PPI) networks, Gene Ontology (GO) analysis, and Kyoto Encyclopedia of Genes and Genomes (KEGG) pathway enrichment analysis. The relationship between active ingredients and key targets was then predicted using molecular docking. Subsequently, an *in vivo* EAMG model was employed to evaluate SS's effects and potential mechanisms against MG.

## 2. Materials and Methods

### 2.1. Screening of Active Drug Ingredients and Corresponding Targets

The following terms were used to retrieve the active compound information of SS from the Traditional Chinese Medicine Systems Pharmacology (TCMSP, https://lsp.nwu.edu.cn/tcmsp.php) database: “Semen Strychni” with an OB (oral availability) ≥30% and DL (similarity of patent medicine) ≥0.18. Subsequently, the PubChem database (https://pubchem.ncbi.nlm.nih.gov/) was used to search for the details of the compounds that were not included in the TCMSP database and to convert all active compounds into the SMILES structural formula. The SMILES structural formula was then imported into the Swiss Target Prediction website. Finally, the biological species were set to Homo sapiens to predict all potential target proteins of the drug's active ingredients.

### 2.2. Target Genes Related to MG

The relevant targets in MG were obtained by searching the Online Mendelian Inheritance in Man (OMIM), GeneCards, DrugBank, and Therapeutic Target Database (TTD) databases with the keyword “Myasthenia Gravis.”

### 2.3. Component-Target Visualization Network Construction

All obtained targets between SS and MG were intersected using a Venn diagram. Then, to create the “active compound-target” network, Cytoscape 3.8.1 was fed all potential SS targets and their corresponding active ingredients.

### 2.4. Construction and Analyses of the PPI Network

The SS and MG shared targets were imported into the STRING database to construct a PPI network. Then, the PPI data were downloaded and stored in a TSV format. In addition, Cytoscape and its CytoNCA plug-in were employed to calculate each target gene's parameters during the core target selection.

### 2.5. GO and KEGG Enrichment Analysis

All core targets were imported into the DAVID database for enrichment analysis of GO and KEGG terms. The GO and KEGG pathway analyses were set to *P* < 0.05 to increase the confidence in the related pathways. The top 10 pathways from the GO analysis were presented in a bar graph. The top 20 pathways from the KEGG analysis were plotted using the R programming language as bubble charts. Cytoscape was used to construct the overall network of KEGG signaling pathways and their corresponding targets.

### 2.6. Molecular Docking

Molecular docking is predominantly employed to validate interactions between target proteins and major active ingredients. The primary active compounds and hub targets of SS and MG were molecularly docked in this study. The SDF-formatted structural files of the key active ingredients were downloaded from the PubChem database and saved. Chem3D software (Cambridgesoft, US) was utilized to convert the SDF format to the PDB format and minimize the energy of the molecules. We obtained the protein's crystal structure by searching the Research of Cooperative Organization for Structural Bioinformatics (RCSB) protein database (https://www.pdb.org/). Pymol (Schrödinger, US) and AutoDock 1.5.6 (Olson Laboratory of Scripps Institute, US) software were used to modify the protein's conformation, including removing ligand and water, hydrogenation, atomic typesetting, and total charge calculation. AutoDock Vina1.1.2 software was used to complete the molecular docking. The PyMol and BIOVIA Discovery Studio 2016 (BIOVIA, France) software packages were utilized to visualize docking results.

### 2.7. Drugs and Reagents

The dried seeds of *Strychnos nux-vomica L* were purchased from SanXiang Chinese Herbal Medicine Co. Ltd. Voucher specimens (*Semen Strychni*, MQZ-2016-002) were deposited in the Second Xiangya Hospital, Central South University. The peptide corresponding to the 97–116 region of the rat AChR subunit, R97–116 (DGDFAIVKFTKVLLDYTGHI), was synthesized by AC Scientific Inc. (Xi'an, China). Rat acetylcholinesterase (ACHE), choline acetyltransferase (CHAT), and acetylcholinesterase antibody (ACHE-ab) ELISA kit were obtained from CUSABIO Biotech CO. Ltd (Wu Han, China). Antibodies against AKT and p-AKT were acquired from Abcam (Cambridge, UK). Anti-MAPK and anti-GAPDH were obtained from Proteintech (Chicago, USA).

SS extraction: Smashed raw SS was extracted in 75% acidic ethanol (pH = 5, 1 : 12, w/v) by refluxing three times for 1h each time. Under hot conditions, the extraction was filtered through a 0.45 *μ*m microporous filter membrane, and the filtrate was combined and concentrated until all ethanol had evaporated. The extract was combined with a 1% CMC-Na solution and the pH was adjusted with 0.5 mol/L NaOH to 6.5 to achieve a 0.05 g raw SS/0.5% ml CMC-Na solution. The contents of brucine and strychnine in the SS extract were determined to be 0.305 and 0.136 mg/ml, respectively.

### 2.8. Animal and Ethical Approval

This study utilized 18 Lewis rats (6 weeks old, 120–138 g). The rats were provided by Beijing Vital River Laboratories (Beijing, China; license number: SCXK (Beijing) 2012-0001). Animals were raised in the barrier facilities in the Department of Laboratory Animals, Central South University [Experimental Animal Use Permit No. SYXK (Xiang) 2015-0017]. The animals were exposed to a controlled temperature environment (24°C–26°C), relative humidity (40%–60%), and a 12/12 h light/dark cycle with free access to food and water for one week. This study was reviewed and approved by the Animal Care and Use Committee of Central South University. All procedures followed the Guide for Care and Use of Laboratory Animals (Chinese Council).

### 2.9. Induction of EAMG and Clinical Evaluation

Randomly, the 18 rats were divided into three groups of six rats each: the control, EAMG, and SS groups. The EAMG model was developed using previously described procedures. We injected 12 rats subcutaneously with 100 *μ*g/200 *μ*L rat 97-116 peptides in Complete Freund's adjuvant (CFA, Sigma) in the waist, abdomen, and hind-foot pads, then boosting with 50 *μ*g rat 97–116 peptides in 200 *μ*l of Incomplete Freund's adjuvant (IFA) on day 11. The control rats were injected at the same sites with CFA emulsified in PBS and IFA in PBS. The SS group received an intraperitoneal injection with SS extraction from day 1 to day 21, whereas the control and EAMG groups received normal saline.

Clinical evaluation in the rat model was scored as follows: according to the EAMG clinical score: 0, no fatigue and normal strength; 1, slightly impaired activity, fatigable, and weak grip; 2, weakness, tremor, head down, and stooped posture before clinical signs; 3, severe weakness, no grip moribund; and 4, death. Animal weights and Lennon scores were measured every day after the final immunization and treatment and every two days after the initial vaccination. On day 22, all rats were sacrificed. During dissection, heart blood was swiftly collected and centrifuged at 3,000 rpm for 10 min. The gastrocnemius muscle was extracted for further analysis.

### 2.10. RNA Isolation and Quantitative Real-Time PCR

According to the network pharmacology results, the mRNA expression levels of CHRM2, CHRNA3, CHRNB4, CHRNB3, CHRM1, CHRNA2, CHRNA4, CHRNA7, CHRNE, MAPK1, AKT1, SLC18A3, ESR1, and MAPK14 were determined using quantitative reverse-transcription polymerase chain reaction (qRT-PCR). Briefly, the TRIzol reagent was used to extract total RNA from muscle tissue. Then, 1 *μ*g total RNA was reverse-transcribed using a reverse transcription kit (Takara, China). Next, utilizing a Bio-rad CFX-96 real-time PCR system (Bio-rad, USA) and a 2X SYBR green qPCR master mix (B21023, Bimake, USA), qRT-PCR was conducted.  Table 1 lists both forward and reverse primers. The qRT-PCR procedure was as follows: 10 min at 95°C (predenaturation), 40 cycles at 95°C for 15 s, and 60°C for 30 s.

### 2.11. Enzyme-Linked Immunosorbent Assay (ELISA)

After centrifugation, the blood supernatant was removed and assayed immediately. The protein expression level of ACHE-ab, ACHE, and CHAT were determined using ELISA kits according to the manufacturer's instructions.

### 2.12. Western Blot Analysis

Total protein was isolated from muscle tissue samples. On 10% SDS-PAGE gel electrophoresis, proteins were separated and transferred to polyvinylidene fluoride (PVDF) membranes. The membranes were blocked for 1.5 h at room temperature in TBST-buffered saline containing 5% (w/v) skim milk and then incubated overnight at 4°C with antigen-specific primary antibodies. The membranes were incubated with anti-MAPK, anti-AKT, anti-p-AKT, and GAPDH primary antibodies, respectively. Blots were then incubated with species-specific secondary antibodies for 60 min. Protein bands were spotted using chemiluminescence detection reagents. Scan densitometry was utilized to quantify the band's intensity. Each measurement was performed at least three times.

### 2.13. Statistical Analysis

Data were presented as mean ± standard deviation (SD). Using SPSS 20.0, the experimental results were statistically analyzed. The One-way analysis of variance was used to compare the differences in experimental data across groups, and the LSD test was then used to compare two groups. *P* < 0.05 was deemed statistically significant.

## 3. Results

### 3.1. Predictive Results of Targets

A total of 13 compounds were achieved from the TCMSP database using the keyword “Semen Strychni” with OB ≥ 30%, DL ≥ 0.18, which was set based on the ADME principle and recommended by the TCMSP database. However, the primary active ingredients of SS, strychnine, and brucine, were not included in the TCMSP screening results. As shown in Table 2, strychnine, brucine, and the other 13 components obtained from TCMSP were selected as potential active components in this study. After standardizing the name *via* the UniProt database and removing duplicates, 388 related targets corresponding to these 15 active ingredients were retrieved from the TCMSP and Swiss Target Prediction websites.

**Figure 1:**
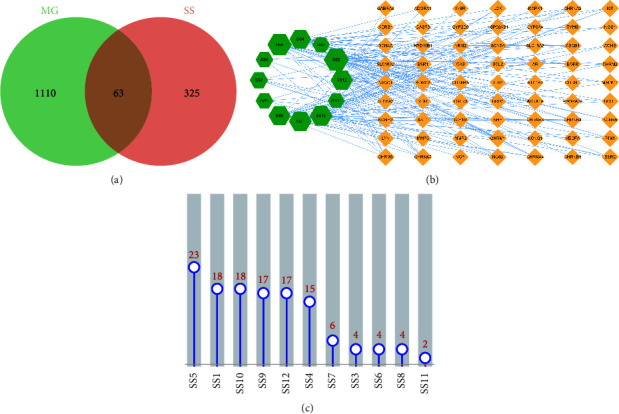
Venn diagram and the active component-target network. (a) Venn diagram of SS- and MG-related targets. Blue circle: targets of MG; pink circle: targets of SS. (b) The active component-target network. The green hexagon represented active ingredients; the yellow diamond represented intersecting targets. The edges represented the connection between active ingredients and targets. (c) Bar chart of targets corresponding to important active ingredients.

Four human gene databases, namely, OMIM, GeneCards, DrugBank, and TTD, were searched using the keyword “Myasthenia Gravis.” Following the elimination of duplicates, 1,173 genes were identified in MG. Drug targets were intersected with the disease targets *via* the Venn diagram (Figure 1(a)). Finally, 63 potential targets were identified, indicating that these targets may have crucial effects on SS used to treat MG.

### 3.2. Network Construction

Cytoscape software was used to create the “drug component-target” network diagram. There were 75 nodes and 153 edges in the network. The green nodes represented the SS's active components. The yellow nodes represented potential MG targets, whereas the edges indicated the relationship between ingredients and targets. As shown in Figure 1(b), most of SS's active ingredients had multiple targets. Similarly, numerous targets corresponded to multiple active ingredients, indicating that SS treated MG *via* a multicomponent and multitarget mechanism. Furthermore, the degree was proportional to the size of the nodes. Higher degrees indicated network nodes with greater importance.  Figure 1(c) depicts the number of targets corresponding to important active ingredients. A total of 12 components of SS have been associated with MG. Isobrucine and vomicine indicated higher degrees and may play crucial roles in treating MG.

**Figure 2:**
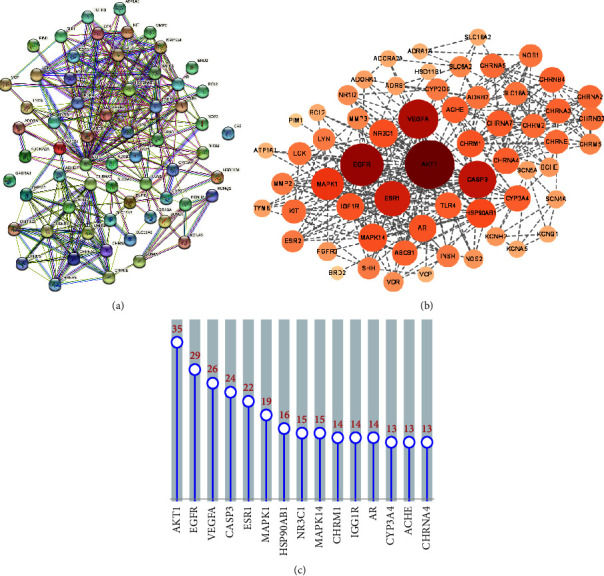
Common-target network. (a) The potential targets of SS preventing MG mapping by string database. (b) The PPI network of potential targets. (c) Bar chart of the top 15 genes according to the degree.

All targets were imported into the STRING online service platform to construct a PPI network, which was then visualized using Cytoscape. As shown in Figures 2(a) and 2(b), the PPI network contained 61 nodes and 312 edges. The nodes represented protein molecules, whereas the edges denoted their relationships. The degree was indicated by the size and intensity of the color (from light to dark). The higher degree values of AKT1, EGFR, VEGFA, CASP3, ESR1, MAPK1, HSP90AB1, NR3C1, MAPK14, CHRM1, IGF1R, AR, CYP3A4, ACHE, and CHRNA4 suggest they may be the primary targets. The degree level of each hub target is displayed in Figure 2(c).

### 3.3. Enrichment Analysis and Target Pathway Analyses

**Figure 3:**
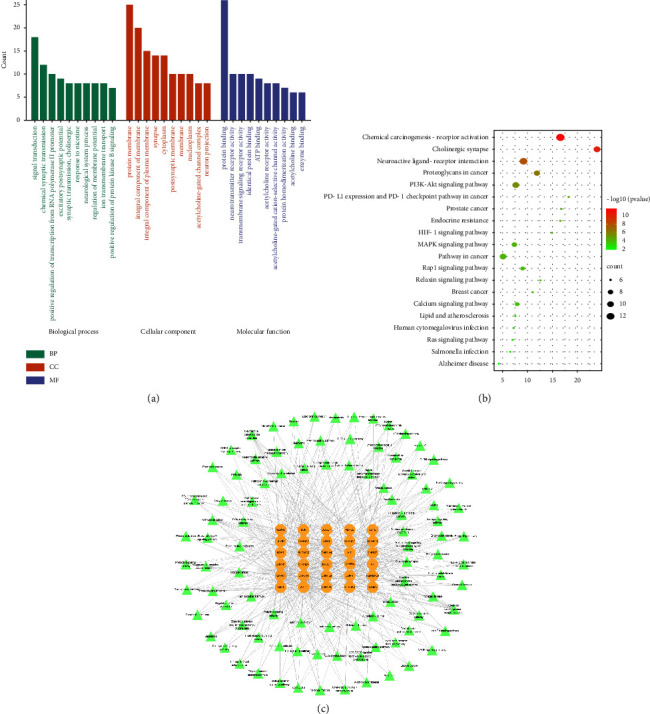
GO and KEGG pathway enrichment analysis. (a) Enriched GO terms; (b) enriched KEGG pathways. The top 10 terms of BP, CC, and MF and the top 20 KEGG signal pathways were selected according to the count. (c) The overall network of KEGG signaling pathways with their corresponding targets. The core target genes were shown as yellow circular, and the pathways were marked with a green triangle.

DAVID 6.8 was used to further analyze the top 30 potential targets to comprehend the underlying mechanisms. A GO enrichment analysis was performed to describe the gene's functions. The GO terms have been categorized as the biological process (BP), cellular component (CC), and molecular function (MF). The top 10 GO terms, from large to small, were imported for visualization into the bioinformation platform (Figure 3(a)). BP was comprised primarily of signal transduction, chemical synaptic transmission, and positive regulation of transcription from RNA polymerase II promoter. CC was involved in the plasma membrane, integral component of membrane, and integral component of the plasma membrane. Protein binding, neurotransmitter receptor activity, and transmembrane signaling receptor activity were primarily associated with MF.

A KEGG pathway enrichment analysis was performed to identify the signal pathways that were significantly enriched. As a result, 94 pathways were obtained in total. According to the *P* value, the top 20 pathways were displayed in an enrichment dot bubble (Figure 3(b)). The top 3 KEGG pathways were the chemical carcinogenesis-receptor activation, neuroactive ligand-receptor interaction, and cholinergic synapse signaling pathway. Based on the literature, we hypothesized that neuroactive ligand-receptor interaction and cholinergic synapse signaling pathway were more likely to be involved in the mechanism of SS in the treatment of MG. Therefore, we constructed a target-pathway network to further deduce the key biological mechanisms. As depicted in Figure 3(c), the neuroactive ligand-receptor interaction pathway was closely associated with CHRM2, CHRNA3, CHRNB4, CHRNB3, CHRM1, CHRNA2, CHRNA5, CHRNA4, CHRNA7, CHRNE, ADRB2, and NR3C1; the cholinergic synapse pathway was mainly related to targets including CHRM2, ACHE, CHRNA3, CHRNB4, CHRM1, CHRNA4, CHRNA7, MAPK1, AKT1, and SLC18A3. In the subsequent molecular docking analysis and *in vivo* experiments, we examined the anti-MG mechanism of SS by focusing on the neuroactive ligand-receptor interaction and the cholinergic synapse pathway.

### 3.4. Molecular Docking Analysis

**Figure 4:**
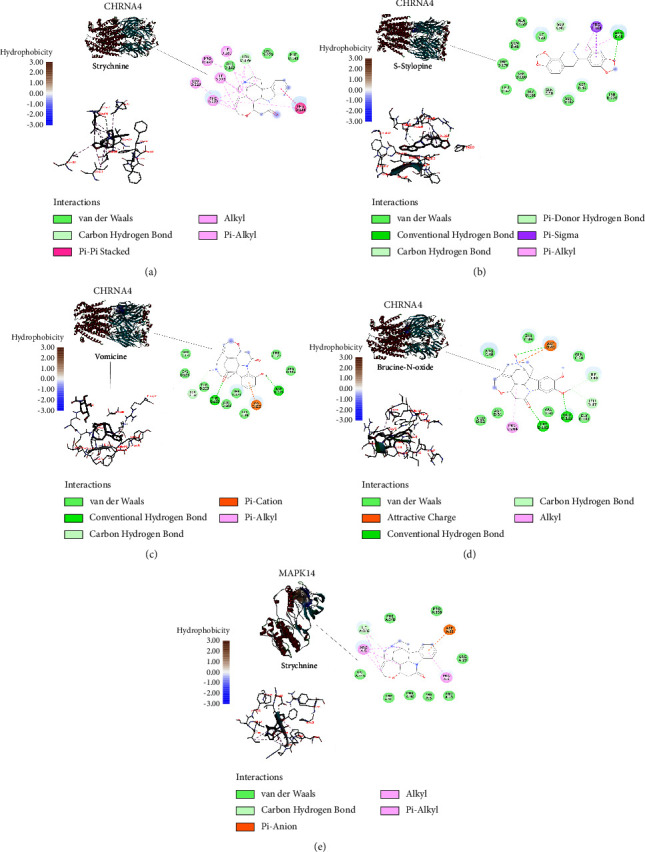
Molecular docking diagram of some active compounds and core targets. (a) Strychnine-MAPK14, (b) strychnine-CHRNA4, (c) vomicine-CHRNA4, (d) (s)-stylopine-CHRNA4, and (e) brucine-N-oxide-CHRNA4. Ligand docked in the binding pocket of protein was placed at the left, the interaction diagram was below, and 2D ligand interaction diagram was shown at the right.

To further validate the network analysis results, molecular docking was performed with a key target (AKT1, MAPK1, MAPK14, CHRM1, ACHE, and CHRNA4) and their respective active compounds (isobrucine, vomicine, (s)-stylopine, strychnine, brucine-N-oxide, and brucine). The docking results of the binding affinity are displayed in Table 3 and visualized using Discovery Studio software. The results for the top five affinities, ranked from the smallest to largest, are exhibited in Figure 4. The remaining results are shown in supplement Figures 1 and 2. The results demonstrated that CHRNA4 binds to the active ingredients most readily. The binding affinity of strychnine to CHRNA4 was the lowest, at −9.7 kcal/mol. Strychnine could form one Pi-Pi Stacke Bond and one hydrogen bond with PHED:144 and LEUD:376, respectively. Besides, it could form Alkyl Bond and Pi-Alkyl with ILED:280, ILED:225, PROD:380, LEUD:229, and PHED:377. Meanwhile, the van der Waals forces between strychnine and CHRNA4 may increase the binding's stability. AKT1 and isobrucine had the weakest binding affinity, with a value of −6.8 kcal/mol.

### 3.5. SS Ameliorated EAMG Symptoms

**Figure 5:**
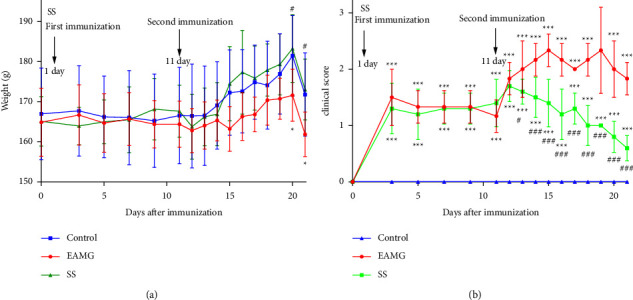
SS treatment ameliorated the severity of EAMG in Lewis rats. (a) The average weight and (b) Lennon scores of rats in the control, EAMG, and SS groups (*n* = 6) were assessed. Data were expressed as the mean ± SD.  ^*∗*^*P* < 0.05,  ^*∗∗*^*P* < 0.01, and  ^*∗∗∗*^*P* < 0.001*versus* the control group; ^#^*P* < 0.05, ^#^*P* < 0.01, and ^###^*P* < 0.001*versus* the EAMG group.

Body mass and Lennon score were used to evaluate the clinical symptoms in the Lewis rats (Figure 5). After modeling, the rats exhibited thinning bodies, muscular weakness, decreased activity, and reduced food intake. The SS intervention alleviated all of the above symptoms. On day 11 after immunization, the average body weight of EAMG rats was obviously less than that of the control group. In addition, the Lennon scores were higher than the control group scores. SS could significantly reverse all the symptoms. The results revealed that SS has a curative effect on EAMG rats.

### 3.6. SS Regulated Cholinergic Synapse and Neuroactive Ligand-Receptor Interaction

**Figure 6:**
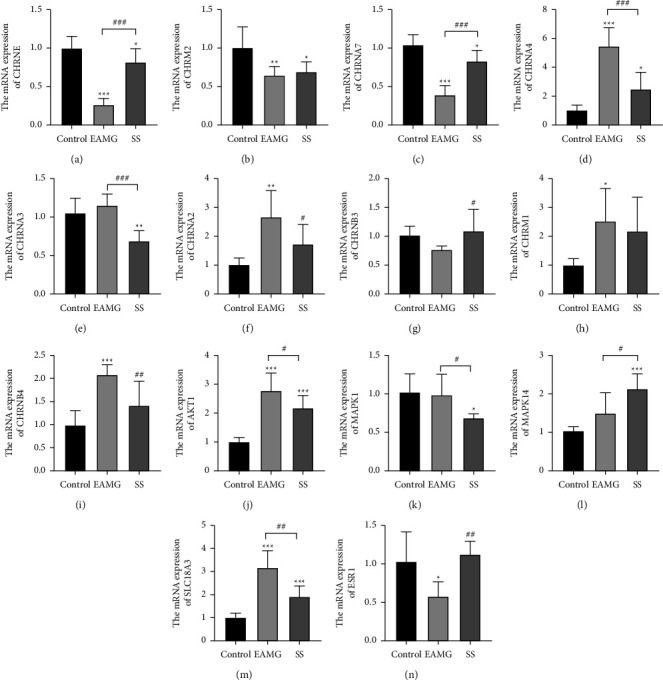
Effect of SS on cholinergic synapse and neuroactive ligand-receptor interaction in the skeletal muscle. The mRNA expression of (a) CHRNE, (b) CHRM2, (c) CHRNA7, (d) CHRNA4, (e) CHRNA3, (f) CHRNA2, (g) CHRNB3, (h) CHRM1, (i) CHRNB4 (j) AKT1, (k) MAPK1, (l) MAPK14, (m) SLC18A3, and (n) ESR1 in each group. Data were presented as mean ± SD.  ^*∗*^*P* < 0.05,  ^*∗∗*^*P* < 0.01, and  ^*∗∗∗*^*P* < 0.001*versus* the control group; ^#^*P* < 0.05, ^#^*P* < 0.01, and ^###^*P* < 0.001*versus* the EAMG group (*n* = 5).

The KEGG pathway enrichment analysis indicated that the cholinergic synapse and the neuroactive ligand-receptor might be the pivotal target of SS to EAMG. Hence, alongside the degree value in the PPI network, the mRNA expressions levels of CHRNE, CHRM2, CHRM1, CHRNA2, CHRNA3, CHRNA4, CHRNA7, CHRNB3, CHRNB4, AKT1, MAPK1, MAPK14, ESR1, and SLC183A in gastrocnemius muscle were detected by qRT-PCR.  Figure 6 demonstrates the results. In the EAMG group, the mRNA expression levels of CHRNE, CHRNA7, and ESR1 were significantly lower than in the control group. SS administration obviously increased the levels of mRNA expression of those genes. Compared to the control group, EAMG increased the expression of CHRNA4, CHRNA2, CHRNB4, AKT1, and SLC18A3, whereas the SS treatment significantly reversed these variations.

### 3.7. SS Regulated the Expression of ACHR-ab, ACHE, and CHAT in EAMG Rats

**Figure 7:**
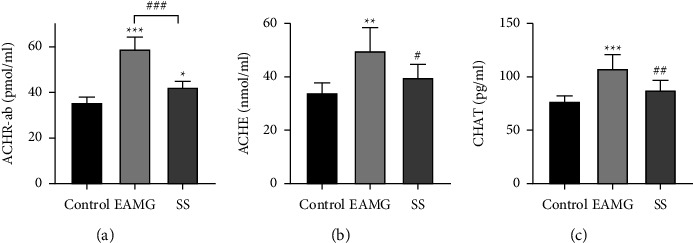
SS regulated serum biochemical parameters in EAMG rats. The levels of ACHR-ab (a), ACHE (b), and CHAT (c) in serum analyzed by ELISA. The results were expressed as mean ± SD.  ^*∗*^*P* < 0.05,  ^*∗∗*^*P* < 0.01, and  ^*∗∗∗*^*P* < 0.001*versus* the control group; ^#^*P* < 0.05, ^#^*P* < 0.01, and ^###^*P* < 0.001*versus* the EAMG group (*n* = 5).

The expression of ACHR-ab, ACHE, and CHAT was measured following the kit's instructions, as performed in Figure 7. Our results demonstrated that the content of ACHR-ab in the EAMG group was significantly higher than that in the control group (*P* < 0.001), and this difference could be reversed following the SS treatment (*P* < 0.001). ACHE is the key hydrolase of ACH. Compared with the control group, the ACHE content in the EAMG group increased significantly (*P* < 0.01), whereas the SS intervention reduced the ACHE content significantly (*P* < 0.05). CHAT is the primary biosynthase of ACH. The concentration of CHAT was significantly elevated in EAMG rats and revised in SS rats. These findings indicated that EAMG affects the cholinergic synapse and that SS treatment could restore choline dysfunction and ameliorate EAMG.

### 3.8. Effects of SS on the Expression of AKT and MAPK in EAMG

**Figure 8:**
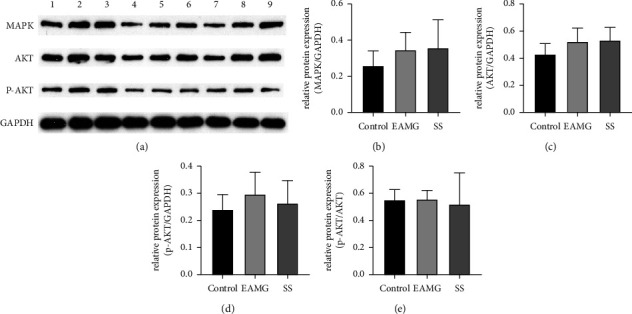
Effect of SS on MAPK and AKT pathway in Lewis rats. (a) The Western blot images of each protein. Representative quantification of (b) MAPK, (c) AKT, (d) p-AKT, and (e) p-AKT/AKT protein levels. GAPDH was used as a loading control.

We investigated whether SS could exert therapeutic effects on EAMG by modulating the protein expression of AKT, p-AKT, or MAPK. As shown in Figure 8, the protein expression of p-AKT was slightly higher in the EAMG group than in the control group. SS marginally decreased p-AKT protein expression in comparison to the EAMG group. The relative protein expression of AKT, p-AKT, and MAPK did not differ significantly among the three groups.

## 4. Discussion

MG is an autoimmune disease caused by neuromuscular transmission retardation, which can be life-threatening and reduce the quality of life. TCM plays a promising role in the treatment of MG. In recent years, there have been an increasing number of reports describing the impact of TCM on MG [4, 13–15]. Clinical practice indicates that SS has an apparent effect on MG. Studies demonstrated that treatment with SS decreased the production of proinflammatory cytokines and inhibited the TLR-4/NF-kB signaling pathway in EAMG rats [9]. Although many studies have focused on the anti-inflammatory and neurotransmitter-regulating effects of SS [16, 17], the underlying mechanisms have not been systematically uncovered. Our team desired to further investigate the mechanism of SS in the treatment of MG.

TCM possesses multicomponent, multipath, and multitarget characteristics, in contrast to conventional chemotherapeutics focusing on a single target. Network pharmacology emphasizes the multichannel regulation of signaling pathways and supplies a potential method for investigating the multitarget mechanism of TCM [18–21]. Through the network pharmacology study, isobrucine, vomicine, (s)-stylopine, strychnine, brucine-N-oxide, and brucine were identified as the most active anti-MG compounds of SS. The principal active and toxic constituents of SS are alkaloids, in which strychnine and brucine comprise 60%–80% of the total alkaloid content [22]. Strychnine, the most abundant alkaloid of SS, is highly toxic to humans and most domestic animals. Strychnine is a well-known potent antagonist of glycine receptors in the central nervous system of vertebrates and a potent blocker of various types of muscle and neuronal nicotinic acetylcholine receptors [23]. Strychnine has negligible antitumor, analgesic, and anti-inflammatory properties [24]. Brucine and brucine N-oxide have been proved to be primarily responsible for the analgesic effects of SS [25]. In addition, brucine N-oxide was ineffective against HepG2 cell proliferation [26]. Numerous studies have shown that brucine and vomicine were potential antitumor compounds [27]. Antioxidant and anticholinesterase assays demonstrated stylopine's beneficial potential [28]. The components of SS that exert the greatest anti-MG effect must be investigated further.

According to the topology data, the SS to MG hub targets were AKT1, EGFR, VEGFA, CASP3, ESR1, MAPK1, HSP90AB1, NR3C1, MAPK14, CHRM1, IGF1R, AR, CYP3A4, ACHE, and CHRNA4. The KEGG enrichment analysis identified chemical carcinogenesis-receptor activation, neuroactive ligand-receptor interaction, and cholinergic synapse signaling pathway as the three most important potential signaling pathways. The results of molecular docking indicated that the major active compounds of SS had a strong binding activity with the hub targets, suggesting that isobrucine, vomicine, (s)-stylopine, strychnine, brucine-N-oxide, and brucine may play an anti-MG role by directly binding to AKT1, MAPK1, MAPK14, CHRM1, ACHE, and CHRNA4. AKT1 plays an essential role in promoting and modulating stress and the apoptosis process. AKT1 encodes a protein member of the serine-threonine protein kinase family and influences numerous biological processes, including metabolism, proliferation, and angiogenesis [29]. According to prior research, the expression levels of AKT1 were significantly elevated in MG patients [30]. Monoclonal anti-ACHR antibodies inhibit AKT phosphorylation in response to insulin, which may contribute to muscle fatigue in MG patients [31]. By regulating immunity, the MAPK signaling pathway contributes to pathological conditions of the nervous system and autoimmune diseases. Competitive endogenous RNA network and pathway-based analysis revealed that the MAPK signaling pathway was essential for lncRNA-SNP-mediated ceRNA regulation pairs associated with MG [32]. The activation of the MAPK signaling pathway affected the activity of various transcription factors and then regulated the expression of tumor necrosis factors (TNF), interleukin-1 (IL-1), interleukin-6 (IL-6), and other inflammatory cells [33]. It was discovered that CHRM1, CHRNA4, and ACHE were all closely associated with choline function. The dysregulation of the cholinergic system and inflammation are involved in the pathophysiology of MG [34].

To further verify the relationship between SS and MG, female Lewis rats were immunized with 97∼116 synthetic rat peptides, and a model of EAMG was successfully induced. Lewis rats were susceptible to MG, which aided in its rapid onset, especially in young female rats [35]. In our study, we discovered that SS treatment ameliorated MG symptoms. SS treatment could improve the weight loss and decrease Lennon scores in EAMG rats, particularly after secondary immunization.

Anti-ACHR antibodies are identified as the leading cause of MG in 80%–90% of the diagnosed patients [36]. Reports revealed that SS decreased ACHR-ab levels in EAMG rats [9]. We also found that SS could reverse the serum ACHR-ab elevation in the EAMG group. ACH is a neurotransmitter and neuromodulator secreted throughout the mammalian cortex to promote cognitive functions, such as learning, memory, and attention [37]. In a normal neuromuscular junction, releasing ACH-containing vesicles into the synapse from the presynaptic terminal activates the postsynaptic ACH receptor and elicits a local depolarization and an end plate potential that is proportional to the amount of ACH binding to the ACH receptor [38, 39]. The reduction in ACH receptors or ACH production in the neuromuscular transmission is closely related to the physiopathology of MG [40]. CHAT, the rate-limiting enzyme that synthesizes ACH, and ACHE, the enzyme that hydrolyzes ACH, were affected by cholinergic disorders [41]. In our study, SS treatment reduced the content of ACHE in EAMG rats. Nonetheless, the CHAT content was significantly upregulated in EAMG rats and altered by SS treatment. It may be due to the negative feedback regulation caused by the decrease in ACH concentration, which increases CHAT expression. The qRT-PCR results indicated that the mRNA expression levels of CHRNE and CHRNA7 were decreased in EAMG rats, whereas the mRNA expression levels of CHRNA4, CHRNA2, CHRNB4, and SL18A3 were elevated. SS could reverse these alterations in mRNA expression. Consistent with network pharmacology and molecular docking results, the results suggested that SS may alleviate MG symptoms by improving choline dysfunction.

The network pharmacology and molecular docking study suggested that AKT1, MAPK1, and MAPK14 were also the hub gene in treating SS against MG. Following the previous study, the mRNA expression levels of AKT1 were elevated in EAMG rats and declined after SS treatment [30]. The slight increase in p-AKT protein expression in EAMG rats suggests that MG may affect the phosphorylation of AKT. However, the protein expression level of AKT, p-AKT, and p-AKT/AKT did not change significantly. It could be due to the small sample size. In our experiment, neither the mRNA nor protein expression levels of MAPK changed noticeably. Therefore, MAPK may not play a significant role in the direct treatment of SS against MG. SS has been shown to have immunomodulatory and anti-inflammatory properties [9]. However, our experiment revealed that SS might play a crucial role in treating EAMG by regulating the function of cholinergic synapses. Additional *in vitro* and clinical studies experiments are required to confirm the detailed mechanisms. Further investigation into how SS binds to the cholinergic synapse to alleviate MG symptoms will contribute to understanding the precise molecular mechanisms and potential treatment targets for MG. By screening the active components of SS, analyzing the target of the main active components, and predicting the mechanism of MG, the study could generate new ideas and directions for targeted intervention therapy in treating MG, ultimately leading to the development of combination drugs.

## 5. Conclusions

In conclusion, we employed network pharmacology and molecular docking techniques to systematically analyze the related targets and signaling pathways of SS against MG. Based on the results of docking studies, the main ingredients, including isobrucine, vomicine, (s)-stylopine, strychnine, brucine-N-oxide, and brucine, were anticipated to bind to and regulate the function of AKT1, MAPK1, MAPK14, CHRM1, ACHE, or CHRNA4, which could be related targets for the treatment of MG. Furthermore, the preliminary *in vivo* experiment demonstrated that SS regulated the cholinergic synapse through the associated antibody, receptor, and key enzymes in the cholinergic pathway. This research could lead to a new understanding of the complex mechanisms underlying TCM's functions and new concepts and directions for targeted intervention therapy in the future treatment of MG.

## Figures and Tables

**Table 1:** Real-time polymerase chain reaction primers.

Target	Forward (5′ to 3′)	Reverse (5′ to 3′)
CHRM1	CTCCCTGTCACGGTCATGTG	CACCTTTGCCTGGTGTCTCA
CHRM2	GAAGGTTCCCAGGAGGAGAGA	GTGGACAATCAACCTTGTGCG
CHRNA3	AGATGGGACCTGTGGCTACT	AGCTTCTTGTGAGGTTGGCA
CHRNB3	ACACTCTGCGCTTGAAAGGAA	TCTGAGGAGTGCGTCTTCGT
CHRNA2	TTCGACCAGCAGAACTGCAA	TCCTTCAGGTCCACTGTCCT
CHRNA4	CAGCTCCCACGTCTCTGTAG	CTCCAACCAGGCCCATAGTC
CHRNA7	TCATCGTGGGCCTCTCTGTA	GGCATTTTGCCACCATCAGG
CHRNE	CTTCTCCTGGCACTCTTTGGC	AGGTTGGTTAGGGTGACCTTG
AKT1	GTCACCTCTGAGACCGACACC	GCCTCCGTTCACTGTCCAC
ERK2 (mapk1)	TCATTCGCTGCAAGATGGAC	TAAATCCCCAGGCAGTGAGCAT
P38 (mapk14)	AGCTTACCGATGACCACGTT	CACGTAGCCGGTCATTTCGTC
SLC18A3	GTGCGAGGACGACTACAACT	GACCTAAATGGGGCGGGTAG
ESR1	TCGATCATTCGAGCACATTCC	CCCTGCTGGTTCAAAAGCG
CHRNB4	CGCCTGGAGCTATCACTGTC	GGCGGTAGTCAGTCCATTCC
GAPDH	ACAGCAACAGGGTGGTGGAC	TTTGAGGGTGCAGCGAACTT

**Table 2:** Main components of *Semen Strychni*.

Sequence number	TCMS number	Compound name	OB (%)	DL
SS10	MOL001040	(2R)-5,7-Dihydroxy-2-(4-hydroxyphenyl)chroman-4-one	42.36	0.21
SS9	MOL001476	(S)-Stylopine	51.15	0.85
—	MOL003410	Ziziphin_qt	66.95	0.62
SS7	MOL003411	Icaride A	48.74	0.43
—	MOL003413	Isostrychnine N-oxide (I)	35.45	0.8
SS6	MOL003414	Isostrychnine N-oxide (II)	37.33	0.8
SS8	MOL003418	Lokundjoside_qt	32.82	0.76
SS5	MOL003432	Vomicine	47.56	0.65
SS4	MOL003433	Brucine-N-oxide	49.17	0.38
SS2	MOL003436	Isobrucine	33.58	0.8
—	MOL003440	Brucine N-oxide	52.63	0.38
SS12	MOL000449	Stigmasterol	43.83	0.76
SS11	MOL000492	(+)-Catechin	54.83	0.24
SS3	MOL003435	Brucine	7.61	0.41
SS1	/	Strychnine	/	/

/: means did not find or have.

**Table 3:** Vina affinity of ingredient-target docking.

Compound	Affinity (kcal/mol)					
	AKT1	MAPK1	MAPK14	CHRM1	ACHE	CHRNA4
Isobrucine	−6.8	/	−7.6	−7.4	/	−8.2
Vomicine	−8.3	/	/	−8.6	/	−9.1
(S)-Stylopine	/	−8.9	/	−8.3	−8.3	−9.6
Strychnine	/	/	−9.1	/	/	−9.7
Brucine-N-oxide	/	/	−8.5	/	/	−9.1
Brucine	/	/	/	−7.5	/	/

/: means did not analyze.

## Data Availability

The data used to support the findings of this study are included within the article.

## Supplementary Materials

Supplementary Materials Supplementary Figures 1 and 2: molecular docking diagram of remaining active compounds and core targets.
