# Supplementary figures and images for: Study on the Potential Mechanism of Semen Strychni against Myasthenia Gravis Based on Network Pharmacology and Molecular Docking with Experimental Verification
Source: Evid Based Complement Alternat Med. 2022 Oct 1;2022:3056802. doi: 10.1155/2022/3056802 (PMC9547686; doi:10.1155/2022/3056802)

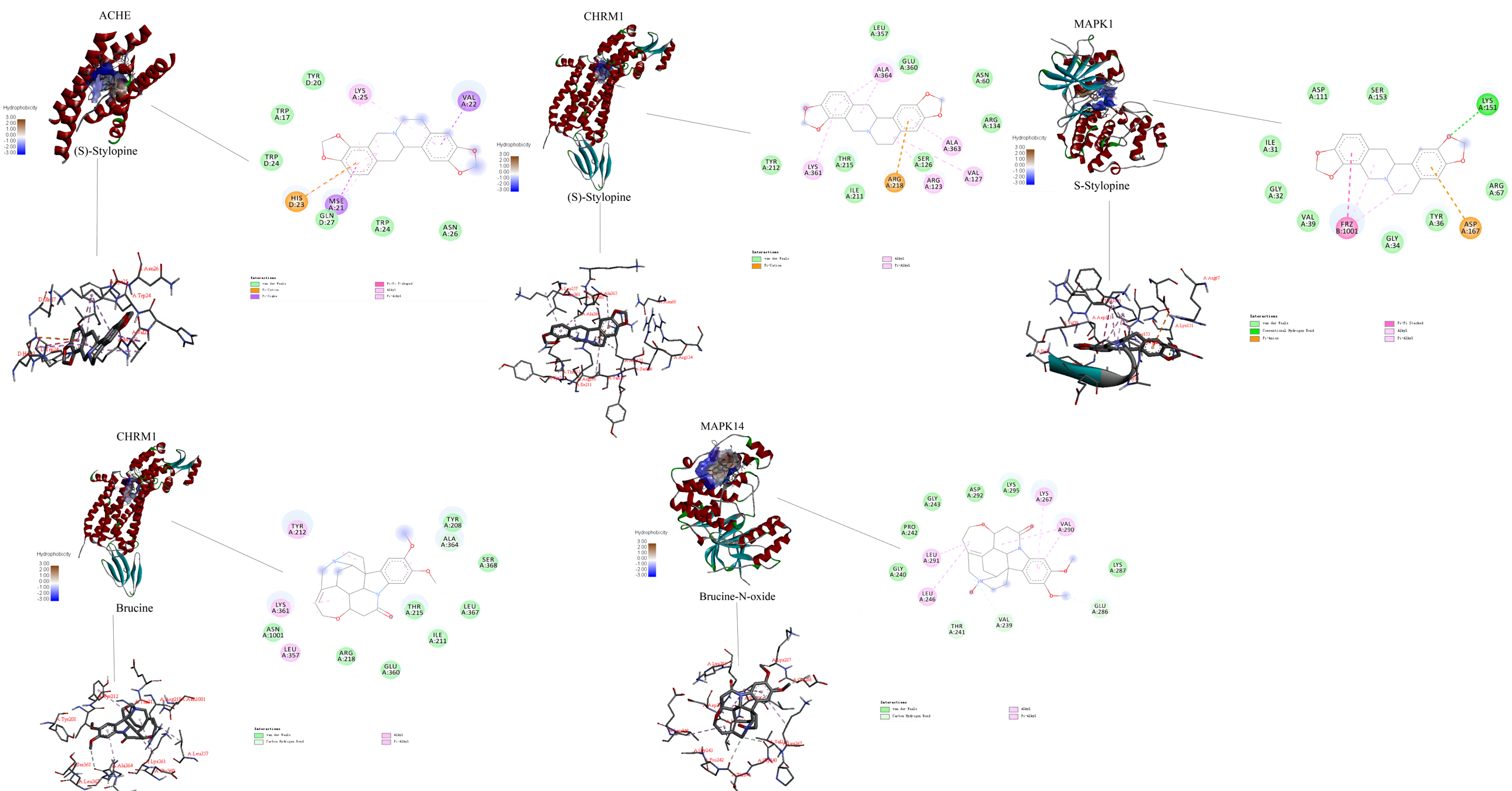

Supplement figure 1: Molecular docking diagram of remaining active compounds and core targets.

Supplement: Supplementary Materials — Supplementary Figures 1 and 2: molecular docking diagram of remaining active compounds and core targets. [file 3056802.f1.zip › supplement figure 1.pdf]

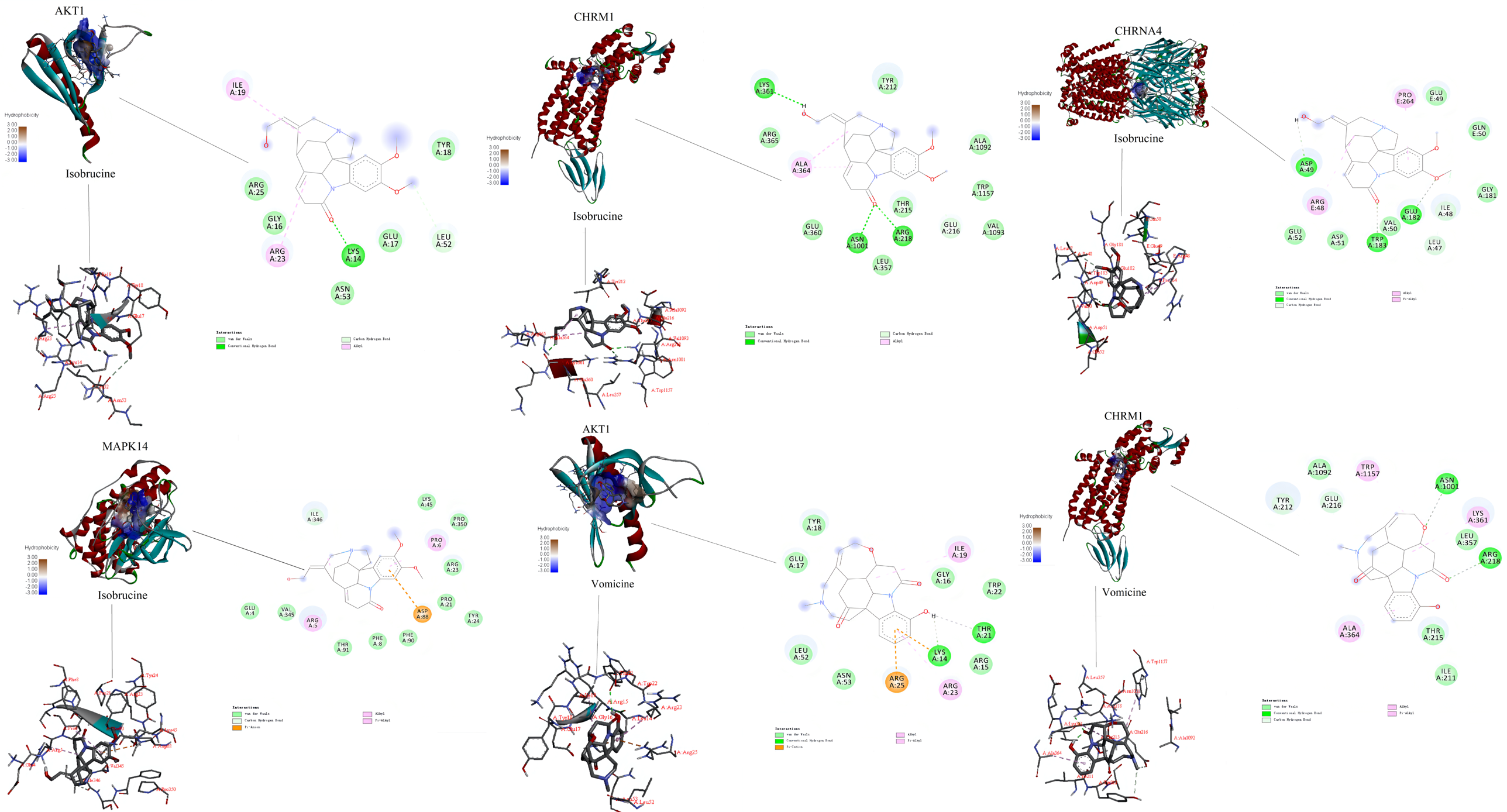

Supplement figure 2: Molecular docking diagram of remaining active compounds and core targets.

Supplement: Supplementary Materials — Supplementary Figures 1 and 2: molecular docking diagram of remaining active compounds and core targets. [file 3056802.f1.zip › supplement figure 2.pdf]
